# Tuberculosis as an Etiological Factor in Liver Abscess in Adults

**DOI:** 10.1155/2016/8479456

**Published:** 2016-08-10

**Authors:** Jaideep Dey, Hitender Gautam, Shwetha Venugopal, Chhavi Porwal, Bijay Ranjan Mirdha, Naresh Gupta, Urvashi B. Singh

**Affiliations:** ^1^Department of Medicine, Lady Hardinge Medical College, New Delhi, India; ^2^Department of Microbiology, All India Institute of Medical Sciences, New Delhi 110029, India; ^3^Department of Medicine, Maulana Azad Medical College, New Delhi, India

## Abstract

*Background*. Tuberculosis of the liver without active pulmonary or miliary tuberculosis is considered as an uncommon diagnosis. The aim of the present study was to determine the etiological role of tuberculosis in adult patients presenting with features of liver abscess.* Methods*. A total of 40 patients with liver abscess were included in the study. The liver abscess aspirate was subjected to microscopy, culture, and polymerase chain reaction to determine the role of tuberculosis as an etiological factor in liver abscess.* Results*. Of the 40 patients enrolled, 25% (10/40) were diagnosed with having tubercular liver abscess. In a total of 40 specimens, 2.5% (1/40) were positive for acid fast bacilli by Ziehl-Neelsen method, while 10% (4/40) were positive for* M. tuberculosis* by culture using BACTEC 460 and the yield increased to 25% (10/40) by polymerase chain reaction for* M. tuberculosis*.* Conclusion*. 25% of the patients presenting with liver abscess had tubercular etiology without features of active pulmonary or miliary tuberculosis. Liver can act as the primary site of involvement in the absence of activity elsewhere in the body. Tuberculosis should be considered as an important differential diagnosis of liver abscess irrespective of evidence of active tuberculosis elsewhere in the body.

## 1. Introduction

Tuberculosis can involve possibly all organs in the human body [1]. Liver is an organ most susceptible to the development of abscesses, caused by numerous organisms, amoebic, bacterial, mycobacterial, or fungal in origin. Liver involvement in pulmonary and extrapulmonary TB is usually clinically silent [2].

Tubercular liver abscess in itself is a rare entity. The disease mimics liver abscess of other etiology, and the diagnosis is frequently missed due to lack of suspicion. Despite reports of involvement of liver in miliary TB, demonstrated in the form of tubercular granulomas in liver tissues [3, 4], local or primary hepatic TB without active pulmonary or miliary TB is considered an uncommon diagnosis [5].

Conventional methods for diagnosis of TB have been ineffective for peritoneal and liver TB; mean sensitivity of culture and AFB smear is 8.3–83% (depending on amount cultured and method utilized) and 3.2%, respectively [6]. In some studies, diagnosis was based on response to empirical antitubercular therapy (ATT) or laparotomy or even autopsy [5]. With increasing reports of resistance to ATT and the morbidity involved with its prolonged usage, response to empirical ATT is an inefficient diagnostic marker.

Liver abscess has traditionally been ascribed to etiologies other than TB [7–10]. The present study was designed to define the role of* Mycobacterium tuberculosis* (M. tb) as the etiological agent for liver abscess in adult patients with no evidence of active TB elsewhere in the body.

## 2. Materials and Methods

Forty consecutive subjects with clinical profile and radiological evidence of liver abscess from outpatient clinics, wards, and emergency departments of medicine at Lady Hardinge Medical College and Dr. Ram Manohar Lohia Hospital, New Delhi, were included in the study. Informed consent was obtained from each participant and clinical history collected.

Patients were excluded from this study if they were hemodynamically unstable, required monitoring in intensive care units, were unable to undergo the requisite workup, and had uncontrolled coagulation defects. The clinical data obtained from each patient included (i) detailed medical history, (ii) clinical features, (iii) hematological laboratory profile like Hb%, leukocyte count, and erythrocyte sedimentation ratio, (iv) liver function test parameters, (v) Mantoux test reactivity, (vi) chest radiography, (vii) ultrasound examination of the abdomen, (viii) stool examination for ova/cyst, and (ix) serological tests for hepatitis B and hepatitis C and Human Immunodeficiency Virus (HIV).

Patients underwent diagnostic ultrasound guided aspiration for liver abscess. The aspirate was subjected to gram stain, bacterial culture, and microscopy for trophozoites and cysts of* Entamoeba histolytica. *The tubercular etiology of the liver abscess was diagnosed by Ziehl-Neelsen (ZN) staining for acid fast bacilli, culture by liquid culture (BACTEC 460), and detection of M. tb DNA using* Mpt64 gene *polymerase chain reaction performed on the liver aspirate.

Chi-square test was used to study the statistical significance of differences between patients with tubercular etiology and nontubercular etiology.

## 3. Results

Of 40 specimens, one (2.5%) was positive for acid fast bacilli by ZN method, while 4/40 (10%) were positive for M. tb by culture using BACTEC 460 (Becton Dickinson) and the yield increased to 10/40 (25%) by PCR for M. tb.

The mean age of the patients in the study was 40.35 ± 13.03 years (mean ± SD) and of the tubercular liver abscess patients was 41.9 ± 13.57 years. Male to female ratio in the study was 4.7 : 1 (82.5% male, 17.5% female) and in tubercular group was 4 : 1. The predominant symptoms in the study, in both tubercular and nontubercular liver abscess patients, were fever and abdominal pain (Table 1).

Twenty percent of the patients in the study but 50% of the patients in tubercular group had past history of TB and had taken ATT (*p* < 0.05) (Table 1). The family history of TB was given by 15% of the patients in the study, 20% in the tubercular group and 13% in nontubercular group. 20% each from the study group, tubercular and nontubercular group had past history of dysentery.

The mean body mass index (BMI) in the study group was 19.93 ± 2.47 kg/m^2^, 20.46 ± 2.99 in tubercular group, and 19.27 ± 2.29 in nontubercular group. Hepatomegaly was present in all, mean 5.45 ± 2.75 cm in the study, 4.84 ± 1.55 in tubercular group and 5.66 ± 3.04 cm in nontubercular group. The liver span in the study group was 16.53 ± 3.42 cm, in tubercular group 16.35 ± 2.54 and in nontubercular group 16.6 ± 3.7.

The laboratory data including the hematological profile and liver function tests did not show statistically significant difference between the tubercular and nontubercular liver abscess patients (Table 2). The mantoux test gave 22.5% positives in the study group and 50% in the tubercular group (*p* < 0.05) (Table 2). Chest roentgenography was abnormal in 8 study patients (20%); only 2 (20%) were in tubercular group while the rest were in nontubercular group.

On ultrasonography of the abdomen, majority of patients (82.5%) had only right lobe liver abscess, 7.5% had only left lobe liver abscess and 10% had abscess involving both the lobes. Stool examination for cysts/trophozoites showed cysts of* Entamoeba histolytica* in one patient who belonged to the tubercular group. Acid fast bacilli were not detected in the stool of any patient.

The viral serological markers for hepatitis B were detected positive in 5% patients in the nontubercular group, while none of the patients were positive for hepatitis C and HIV. The aspirated pus of all the patients was negative using Gram stain, bacterial culture, and microscopy for trophozoites or cysts of* Entamoeba histolytica*.

## 4. Discussion

Smear examination for acid fast bacilli and conventional culture techniques are the widely used methods of TB diagnosis. Acid fast smears in hepatic TB have shown higher frequency among miliary TB patients [4, 11]. Yamaguchi and Braunstein have shown higher yield with fluorescent staining techniques [12]. In a recent study, sensitivity of conventional methods was 12% and that of PCR assay was 88% [13]. PCR assay increased the sensitivity of detection of M. tb among patients with hepatic granulomas. In the present study while the PCR detected 25% of liver abscess as tubercular, liquid culture and ZN staining could detect only 10% and 2.5%, respectively. More sensitive tests hence hold promise for increasing the yield of diagnosis.

Fever and abdominal pain were the predominant symptoms in the study and tubercular liver abscess group. The other symptoms were weight loss and jaundice, similar to previous studies in hepatic TB [3–5, 14, 15]. The past history of TB and history of previous treatment with ATT were statistically significant in the tubercular liver abscess group (Table 1). Careful history taking may provide a clue to the diagnosis of tubercular liver abscess.

On clinical examination, hepatomegaly was present in all the patients in the study; other studies [3–5, 14, 15] have shown hepatomegaly in 80–96% of patients with hepatic TB. The mean liver span was similar in the tubercular and nontubercular groups.

The liver function tests, though nonspecific, have been used to screen patients for hepatic TB. Some authors have shown raised serum-alkaline phosphatase (SAP) as suggestive of hepatic TB [4, 16], while others have shown presence of normal levels does not rule out hepatic TB [17]. In the present study, raised SAP was seen in 100% of tubercular group and 93% of the nontubercular group. This strengthens the view that abnormal SAP values should be considered with discretion. Serum levels of liver enzymes such as aspartate aminotransferase and alanine aminotransferase were raised in 80% in tubercular group; others have reported elevated levels in 70% [4]. Abnormalities in these enzyme levels in hepatic TB patients were present in 91–94% of jaundiced group and in only 5% of the nonjaundiced patients [18]. Other parameters like serum albumin, serum globulin, albumin/globulin ratio, and gamma-glutamyl transpeptidase were considered in some of the studies [4, 5, 15] but are nonspecific for hepatic TB. Thus abnormalities in liver function test parameters cannot be relied on for the diagnosis of hepatic TB.

In the present study, Mantoux test was positive in 22.5% and was statistically significant among tubercular group when compared with nontubercular group (Table 2). Oliva et al. in his review stated that out of 15 cases of isolated tuberculoma and tubercular liver abscess, 11 had positive reactions of tuberculin skin test whereas two patients were negative and two were anergic [14]. Guckian and Perry reported 41% patients were positive for tuberculin skin test [18].

Chest radiograph in the present study showed abnormalities in 20% study patients; only two belonged to the tubercular group; other studies have also reported normal chest X-rays in patients with local hepatic TB [19–23]. Some authors have shown abnormal chest X-ray in 65–78% in hepatic TB [4, 5, 15]. Essop et al. have published chest radiograph abnormality in 86% patients with acute (miliary) TB, while 25% of chest X-rays in chronic hepatic TB and all of the radiographs in local hepatic TB were normal [4]. Thus diagnosis cannot be excluded based on the normal chest radiograph.

The hypoechoic lesions and complex masses shown in ultrasound of the liver could not differentiate between liver abscess and carcinoma [24–28]. Though hypoechoic lesions are commonly seen in tubercular liver abscess, rarely hyperechoic lesions are also described in the literature [29]. In the present study, right lobe involvement was predominant and there was no statistically significant difference in the pattern of the abscess between tubercular and nontubercular group. Other imaging techniques like computed tomography of the abdomen, technetium colloid liver scans, and abdomen radiography are poorly suggestive of hepatic TB [4, 5, 15].

The study lent credence to the hypothesis that liver abscess can have etiologies other than* Entamoeba histolytica* causing amebiasis. One-quarter of the study patients were clearly shown to have TB as the only etiology of the liver abscess; the abscess probably develops from coalescing caseating nodules as described by Oliva et al. [14]. The study sums up that a patient presenting with symptoms of fever with abdominal pain in addition to an enlarged liver, with or without complaints of weight loss and usually without jaundice, along with a positive Mantoux test and a past history of TB should raise the suspicion of tubercular liver involvement even in the absence of chest/systemic involvement especially in a country with high prevalence of tuberculosis like India, which has one-fourth of the global incident TB cases, estimated annual incidence of 2.2 million TB cases, prevalence of 2.5 million, and annual mortality of 0.22 million, respectively [30]. It is important to keep the differential diagnosis of TB in order to reach the correct etiological diagnosis of liver abscess and instituting early treatment.

## Figures and Tables

**Table 1:** Presenting symptoms and history of patients in the study group.

Symptoms	Overall study group (*n* = 40)	Tubercular group (*n* = 10)	Nontubercular group (*n* = 30)	*p* value
Fever	92.5%	100%	90%	*p* > 0.05%
Abdominal pain	92.5%	100%	90%	*p* > 0.05%
Weight loss	44.5%	30%	53%	*p* > 0.05%
Jaundice	17.5%	0%	23%	*p* > 0.05%
Past h/o tuberculosis	20%	50%	10%	*p* < 0.05

**Table 2:** Laboratory profile of patients.

	Overall study group (*n* = 40)	Tubercular group (*n* = 10)	Nontubercular group (*n* = 30)	*p* value
Hb (<10 g/dL)	75%	88%	70%	*p* > 0.05
Leucocytosis	78%	80%	77%	*p* > 0.05
Raised ESR	85%	90%	83%	*p* > 0.05
Raised serum bilirubin	20%	10%	23%	*p* > 0.05
Raised serum liver enzymes	70%	80%	67%	*p* > 0.05
Raised alkaline phosphatase	95%	100%	93%	*p* > 0.05
Deranged coagulation profile	40%	40%	13%	*p* > 0.05
Mantoux test positive	23%	50%	13%	*p* < 0.05
